# DNA methylation patterns associated with konzo in Sub-Saharan Africa

**DOI:** 10.1186/s13148-022-01372-x

**Published:** 2022-12-19

**Authors:** Kristen Kocher, Surajit Bhattacharya, Matthew S. Bramble, Daniel Okitundu-Luwa, Dieudonne Mumba Ngoyi, Desire Tshala-Katumbay, Eric Vilain

**Affiliations:** 1 Department of Genomics and Precision Medicine, The George Washington University School of Medicine and Health Sciences, Washington, DC 20037 USA; 2 Department of Genetic Medicine Research, Children’s Research Institute, Childrens National Hospital, Washington, DC 20010 USA; 3 Department of Neurology, University of Kinshasa, Kinshasa, Democratic Republic of the Congo; 4 National Institute of Biomedical Research, Kinshasa, Democratic Republic of the Congo; 5 Department of Neurology, School of Medicine, Oregon Health and Science University, Portland, OR 97239 USA; 6 Institute for Clinical and Translational Science, University of California, Irvine, Irvine, CA 92697 USA

**Keywords:** Konzo, Epigenetics, DNA methylation, Cassava, Cyanide, Sub-Saharan Africa

## Abstract

Konzo, a disease characterized by sudden, irreversible spastic paraparesis, affecting up to 10% of the population in some regions of Sub-Saharan Africa during outbreaks, is strongly associated with dietary exposure to cyanogenic bitter cassava. The molecular mechanisms underlying the development of konzo remain largely unknown. Here, through an analysis of 16 individuals with konzo and matched healthy controls from the same outbreak zones, we identified 117 differentially methylated loci involved in numerous biological processes that may identify cyanogenic-sensitive regions of the genome, providing the first study of epigenomic alterations associated with a clinical phenotype of konzo.

**Supplementary Information:**

The online version contains supplementary material available at 10.1186/s13148-022-01372-x.

## Introduction

The World Health Organization (WHO) classifies konzo as a neurological disease characterized by sudden onset of spastic paraparesis in a formerly healthy individual and strongly correlated with the monotonous consumption of insufficiently processed bitter cassava *(Manihot esculenta Crantz*) and malnutrition, specifically a diet deficient in sulfur amino acids [1–4]. Cassava is a fibrous tuber and the predominant food source for many regions throughout Sub-Saharan Africa, including Angola, Cameroon, Central African Republic, Democratic Republic of the Congo (DRC), Mozambique, United Republic of Tanzania, and Zambia, which are the only countries in the world where konzo is endemic [1, 3, 4]. In addition to konzo, consumption of cyanogenic cassava has also been associated with other complex neurodegenerative syndromes in Sub-Saharan Africa, including tropical ataxic neuropathy (TAN) and motor neuron-cerebellar-Parkinson-dementia syndrome [1, 5, 6]. Konzo is predominantly linked to rural regions in the seven aforementioned countries in Sub-Saharan Africa that lack healthcare infrastructure and resources [1, 3, 4]. The mean annual incidence rate of konzo diagnosis is 0.9 per 100,000 [1]. Studies have determined that cyanide exposure from cassava is associated with a loss of approximately 2 disability adjusted life years and an overall case-fatality ratio of about 21%, making konzo among the most prevalent of any disease associated with chemical exposure through the food supply [1].

Konzo onset typically occurs in a formerly healthy individual, beginning with bilateral spastic movements of the lower limbs that affect gait and progressing to exaggerated knee and ankle spasms, and occasionally full lower limb paralysis [2, 5, 7]. The disease is non-progressive and irreversible, without any apparent pathology of the spinal cord [2, 7]. Konzo can present in a spectrum of severities and occur with comorbidities, such as cognitive impairment [6, 7]. Epidemiological surveys have highlighted that children and women of child-bearing age appear to be more vulnerable to developing konzo, and familial clusters of disease occur for reasons that have yet to be fully elucidated [8].

Uncovering the molecular underpinnings of konzo etiology and its comorbidities are of considerable interest, as cassava is a dietary staple for over 800 million people due to its drought tolerance [1]. All cultivars of cassava have an innately high concentration of the cyanogenic glucosides, predominantly linamarin, which assist in defending the tuber from animals and insects [1]. Proper processing of cassava makes it safe to consume and is crucial to reducing the concentration of linamarin and downstream cyanide exposure [1, 6]. During times of drought, famine, and war, when resources and time are scarce, proper processing techniques diminish and epidemics of konzo become rampant [1, 6].

As konzo can affect up to 10% of the population in regions throughout Sub-Saharan Africa that subsist on a homogenous diet of cassava, it is of critical importance to elucidate the pathophysiologic factors that contribute to disease susceptibility and onset [5]. As epigenetics are known to be heavily influenced by the environment, age, and diet, this study focuses on detecting the underlying epigenomic signatures in individuals in areas of konzo-outbreaks compared to age-matched and sex-matched, unaffected controls from the same outbreak zones in the DRC. DNA methylation has been shown to be a genomic marker of exposure to certain chemicals and thus can be used as a diagnostic signature for poorly understood, environment-induced diseases [9]. By highlighting loci within the genome that are differentially methylated we will be able to better understand the individual susceptibility risks of konzo. This is the first study focused on understanding the underlying epigenomic signatures, or biomarkers, associated with konzo. Future studies should aim to focus on functional validation of the apparent molecular differences highlighted in this study, including extent of dietary exposure, toxicity, and adverse outcome pathways, and explore the complex epigenetic and transcriptional landscape associated with sub-lethal cyanide exposure and the clinical konzo phenotype.

## Methods

### Patient samples

Samples from the konzo outbreak regions of Kahemba, southern Bandundu Province, DRC, were obtained in October to November 2011; samples from the konzo outbreak region in of zone de sane de Mwana, South Kivu Province, were collected in November 2012. From a previously established konzo WHO surveillance list of outbreaks in Kahemba and South Kivu, subjects were identified and used for this study [10]. The institutional review board at Oregon Health and Sciences University and the Democratic Republic of the Congo Ministry of Health provided study approval. Enrolled children were consented by caregivers orally in the local language with signature or thumb print for those not literate. Individuals were phenotypically diagnosed using neurological exam performed by neurologists to confirm konzo diagnosis using the 1996 WHO criteria. For konzo-affected individuals, konzo severity was described as “mildly abnormal (periodic or mild hyperreflexia and exaggerated clonus or reflexive delay)”. Patient demographic data can be found in Additional file 1: Table S1**.**

Whole blood was collected in EDTA tubes from healthy and konzo-affected individuals by local medical staff at two sites in DRC (Kahemba and South Kivu) and separated into serum, buffy coat, and plasma at the collection site and then stored in liquid nitrogen until DNA processing. DNA was extracted from buffy coat and stored at − 80 °C in the Tshala-Katumbay lab (University of Kinshasa, DRC). Samples were transported for long-term storage to the Tshala-Katumbay lab at Oregon Health & Science University (Portland, OR, USA), until they were used for DNA methylation experiments (*n* = 32) and transferred to Children’s National Research Institute (Washington DC, USA).

### Genome-wide DNA methylation array

DNA was quantified using the Qubit dsDNA Broad Range Assay kit and Qubit 4 fluorometer (Thermo Fisher). 300 ng of extracted DNA from each sample was bisulfite converted using the EZ DNA Methylation-Lightning kit (Zymo). Samples were whole-genome amplified, enzymatically fragmented, and hybridized to BeadChip array using the Infinium MethylationEPIC BeadChip kit according to the manufacturer’s protocol (Illumina). Hybridized methylation arrays were scanned using the iScan system (Illumina) at Georgetown University’s Genomics & Epigenomics Shared Resource Core in Washington, DC. The Gene Expression Omnibus accession number is GSE18011.

### Methylation array analysis

**Fig. 1:**
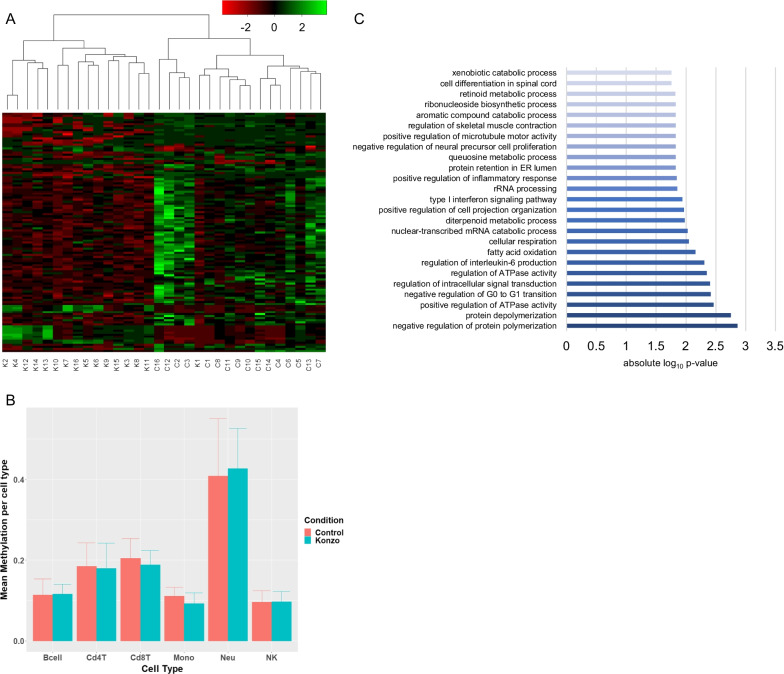
Differentially methylated probes between konzo and control samples reveal unique epigenetic signatures that may relate to clinical presentation and etiology of konzo. **A** Heat Map of DMPs, in beta values, between Konzo Cases and Controls. *Limma* function in R was used to perform linear regression analysis on normalized beta values between konzo cases and age-matched healthy controls. Significant DMPs were determined using a threshold FDR *p* value of < 0.05 and log2 fold-change cutoff of < − 1 or > 1. Z-scores used for scaling for ease in visualization. **B** Box-and-whisker plot visualization of mean methylation per cell type: B cell (Bcell), Cd4 T cell (Cd4T), Cd8 T cell (Cd8T), monocyte (Mono), neutrophil (Neu), and natural killer cells (NK). This analysis was performed for each condition, Konzo (blue) and Control (red). Wilcoxon test was performed and are not statistically significant (*p* ≥ 0.05). Whiskers represent the upper and lower bounds of the mean ± standard deviation. **C** Gene Ontology enrichment (GO) analysis of DMPs found in promoter regions (transcription start sites or 5′ UTR) associated with konzo. Significant GO terms were determined by a *p* value cutoff of < 0.05. Most enriched values were determined using absolute log10 *p* value

Raw intensity values (idat files) were generated from each EPIC BeadChip row using the iScan system. All statistical analysis was done using R (version 4.0.1) and Bioconductor packages. Intensity values were converted to beta (*β*) and *m* values using the Bioconductor *minfi* package. *β* values (*β* = M/(M + U + 100), where *M* is methylated intensity and U is unmethylated intensity for the same position) are used for visualization and *m* value (*M* = log2(*M*/*U*)) for statistical analyses (difference in average konzo *β* values = 0.29; difference in average control *β* values = 0.35; Additional file 1: Table S2). Quality filtration parameters, used to filter out low quality probes, were probes that are represented by less than 3 beads for greater than 5% of the samples (49,632 probes), probes having quality *p* value less than 0.05 (15,032 probes), probes having SNP (9956 probes), and probes mapping to multiple sites in the genome (“cross-reactive probes”; 41,482 probes). Probes filtered for quality *p* values and SNP probes were determined by *detectionP* and *dropLociWithSnps* functions of the *minfi* package, cross-reactive probes by *dropXreactiveLoci* function of the *maxprobes* package and bead related filtration by *beadcount* function of the *wateRmelon* package 9. The probe intensity was normalized by Subset-quantile Within Array Normalization (SWAN). A total of 795,169 quality filtered normalized probes were used for subsequent differential methylation analysis using *limma* package in R. Differential DNA methylation analysis was performed using a statistical threshold cutoff FDR *p* ≤ 0.05 and log2 fold-change cutoff of < − 1 or > 1 (Additional file 1: Table S4). Quality visualization was done using *champ.QC* function of *ChAMP* package in R, and PCA plots were done using base R package. Tool information and references can be found in Additional file 1: Table S3. Data quality plots can be found in Additional file 2: Fig. S1. Sex-prediction analysis did correctly predict the sexes of all but one sample, K16. Additionally, we performed immune cell variation analysis using *FlowSorted.Blood.EPIC* package to determine any potential involvement of immune cell fractions in this analysis by calculating the means per cell type and cohort (Fig. 1C, Additional file 1: Table S5 and S6) and sex-prediction analysis through *minfi*. There were no immune cell fraction differences between konzo cases and controls and the resultant gene list contained 76% of the resultant genes from the analysis prior to performing immune cell fraction analysis.

### Gene ontology enrichment analysis

Functional annotation was done using Enrichr (https://maayanlab.cloud/Enrichr/) on all differentially methylated probes that appeared in promoter region (defined as either located in the transcription start site (TSS) or 5′ UTR) choosing only gene ontology terms enriched for “Biological Processes” with a statistical significance of *p* ≤ 0.05. Duplicated or redundant gene ontology terms were reduced through the REVIGO interface using standard parameters and visualized using GOplot (details in Additional file 1: Table S7).

### Validation of array-based DNA methylation signatures

A targeted next-generation bisulfite sequencing panel was chosen based on 33 probes (Illumina IDs) that were noted to be differentially methylated between cases and controls from our array analysis and used to validate our findings (EpiGenDx). For validation, we chose 8 cases and 8 control samples from our original array analysis and 33 previously identified probe locations of differential methylation (Additional file 1: Table S8). We shipped eluted DNA to EpiGenDx, where they performed bisulfite modification, multiplex PCR, library preparation, and sequencing (Ion Torrent S5). FASTQ files were aligned with Bismark Bisulfite Read Mapper program (v0.12.2) and Bowtie2 (v2.2.3), and Bismark was used to calculate methylation levels. T test was performed to identify difference between the 2 conditions, with significance thresholds.

## Results and discussion

Comparative analysis of normalized intensities derived from interrogation of over 850,000 methylation probe sites between konzo cases and age- and sex-matched healthy controls suggests that there are 117 differentially methylated probes (DMPs) significantly associated with konzo-affected individuals (Fig. 1A). Of the 117 total sites of differential methylation, 99 DMPs were hypomethylated, while 18 sites were hypermethylated compared to controls (Additional file 1: Table S4). The EPIC array data were validated using next-generation targeted bisulfite sequencing, which confirmed our findings at 33 of the most differentially methylated sites (Additional file 1: Table S3). Unsupervised hierarchical clustering of DMP intensities revealed that samples cluster strongly by cohort, except for one sample (K1), which appears to cluster more with the control cohort than konzo (Fig. 1A). Without other clinical information, it is not possible to determine if this sample is clustering differently due to other phenotypic associations (i.e., disease onset or severity) or a true outlier of the study. We did however rule out the influence of immune-cell-type contributions, which were not observed to be significantly different in our analysis (Fig. 1B).

Since there are apparent sex-specific differences in the manifestation and presentation of konzo, where females of child-bearing age appear to be more vulnerable than males (although this may be attributed to social differences), we interrogated konzo males versus konzo females and did not identify significantly differentially methylated loci attributed to sex (data not shown) [7]. However, the analyzed cohort is small and may not have had enough statistical power to uncover sex-specific differences in DNA methylation associated with disease [7].

Of the 117 DMPs between konzo and controls, there were 2 genes with multiple differentially methylated probes that were significantly differentially methylated in the konzo cohort, *ZNF718* and *AKAP12* (Additional file 1: Table S4). All sites associated with the genes *ZNF718* and *AKAP12* were significantly hypermethylated (FDR *p* value ≤ 0.05 and log_2_ fold-change in DNA methylation intensity ≤ 1) in konzo cases, compared to controls (Additional file 1: Table S4). 46 of these sites associated with genes were identified in the promoter region (TSS or 5′ UTR). These 46 sites were analyzed for gene ontology (GO) enrichment at a statistical threshold of *p* ≤ 0.05 and we were able to ascertain that the konzo cohort was enriched for biological processes relevant to konzo etiology and potentially relevant pathways (Fig. 1C, Additional file 1: Table S7). For example, among the top enriched terms, we noted *regulation of skeletal muscle contraction* (GO: 0014819), which may be directly relevant to the spastic movements and paraparesis that are characteristically associated with the konzo phenotype (Fig. 1C) [2]. Additionally, using the Online Mendelian Inheritance of Man database, we identified that the associated gene for this GO term, *KCNJ2*, has been implicated in other disorders of periodic paralysis, such as Andersen Syndrome, and thus may be directly relevant to konzo disease presentation [11].

Of additional interest, we noted significant enrichment for the biological process *queuosine metabolic process* (GO: 0046116). Queuosine is a modified nucleoside present in certain mammalian tRNAs and its abundance has been linked to the presence of micronutrients derived from the gut microbiome and directly links to transcriptional regulation [12, 13]. As konzo onset is linked not only to dietary exposure to cyanogenic glucosides, but also to SAA deficiency, an adjunct role of the gut microbiome could also play into the disease phenotype and be linked to changes in DNA methylation, transcription, and metabolic processes. The literature suggests that DNA methylation is strongly influenced by the environment, so changes in diet that are known to be associated with disruptions in molecular processes and the gut microbiome, like queuosine metabolism, may be of interest for elucidating the complex mechanisms associated with konzo disease onset and progression.

Overall, while enrichment of these biological processes may suggest a role for modifications to DNA methylation in disease phenotype, these 117 sites of differential methylation may serve as biomarkers for monitoring populations at-risk for konzo. Functional validation is critical to further explore these findings and understand the impact of these differentially methylated sites in the context of dietary cyanogenic glucoside exposure and konzo presentation, as well as determine if there are specific epigenetic markers associated with susceptibility or risk to developing a clinical phenotype, as konzo does not present in all who are exposed to the same, homogenous diet of cyanogenic cassava.

A limitation of this study is the small sample size (*n* = 32) that was used. While we were able to determine statistically significant DNA methylation differences between our cohorts, future studies should look to expand on the size of the cohort used, increase the age range, and include numerous disease severity levels, and ensure that a sufficient sample size is used for a well-powered study. Additionally, by correlating the level of in vivo cyanogenic glucoside metabolites in serum or urine at the time of collection could provide invaluable information regarding the level exposure of each individual to cyanogenic cassava through the diet and further be associated with the DNA methylation changes present. As previously mentioned, children are also at high-risk groups for developing konzo. In this cohort, the median age of recruited konzo and healthy control individuals was approximately 13 years old. As such, we are unable to draw conclusions regarding the DNA methylation patterns associated with pediatric versus adolescent ages groups. Future research should aim to observe longitudinal progression of this disease and consider timing of onset and severity of these age groups, which may elucidate the molecular underpinnings of the sudden and irreversible phenotype associated with konzo.

This study has provided the first analysis of epigenetic changes associated with clinical diagnosis of konzo. Future experiments should focus on further identifying biomarkers of low-dose dietary cyanide exposure and identifying factors of konzo disease susceptibility and pathobiology through other molecular approaches.

## Supplementary Information

**Additional file 1**: **Table S1**. Sample collection information. Sample IDs were relabeled with "Analysis ID" for improved readability on figures. Cohort specific information, location of sample collection, and age are also provided (average age = 13 years old). **Table S2**. Beta values of all konzo case and control samples. Beta values for individual konzo samples are labeled columns K1–K16; beta values for individual control samples are labeled C1–C16. Averages for both groups are labeled Average_Konzo_Bvalue and Average_Contrl_Bvalue. Mean for each group is labeled Average_Konzo_Bvalue_Total and Average_Contrl_Bvalue_Total. **Table S3**. DNA Methylation analysis tools, functions and references. **Table S4**. Output significantly differentially methylated probes from bioinformatic analysis. Threshold for significance: FDR *p* value < 0.05, log2 fold-change of less than or equal to − 1 or greater than or equal to 1. Column headers include the Illumina ID (IlmnID), Chromosome Number (Chromosome) and location (Coordinate_start or Coordinate_end), positive or negative strand (Strand), gene name (Name), location of probe to CpG (Group), log2 fold-change value (logFC), and FDR adjusted *p* value (adj.P.Val). **Table S5**. Output significantly differentially methylated probes from bioinformatic analysis with immune cell fraction analysis. Threshold for significance: FDR *p* value < 0.05, log2 fold-change of less than or equal to − 1 or greater than or equal to 1. Column headers include the Illumina ID (IlmnID), Chromosome Number (Chromosome) and location (Coordinate_start or Coordinate_end), positive or negative strand (Strand), gene name (Name), location of probe to CpG (Group), log2 fold-change value (logFC), and FDR adjusted *p* value (adj.P.Val). **Table S6**. Cell-type fraction means for Konzo cases and controls. Standard deviation (sd) was calculated for each mean. Column headers include: cell type (CellType), cohort specifics (Condition), mean (Mean), standard deviation (sd), and Upper (mean + sd) and Lower (mean-sd) bounds. **Table S7**. Output significantly enriched GO Terms. Threshold for significance: *p* value < 0.05. Column headers include the GO ID (ID), GO Term Description (GO Description), absolute log10 *p* value (abs_log10_pval), log10 *p* value (log10_pval), *p* value (pval), and genes associated with GO ID (genes). **Table S8**. Targeted methylation analysis results. ProbeID: Illumina probe IDs; Genes: Associated genes; Transcripts: Associated transcripts; Chromosome: chromosomal position; Region: Regions in the chromosome that get affected; %MethylationDifference: Difference in methylation percentage; logFC: Difference in log (base 2) fold change; *p* value: associated *p* value.

**Additional file 2**: **Fig. S1**. Principal component analysis (PCA) plots show similarity in signal intensity between konzo and control samples. **A** PCA plot for signal intensity of all control and konzo samples before normalization: Principal component analysis done on more than 850 K probes (866,087 probes), reveals a tighter overlap of intensities (principal components) between Konzo samples (blue) compared to red (Control) samples. There is some overlap with between the controls and the Konzo sample, which might lead to the assumption that there is not a huge difference in the epigenetic patterns between konzo and control samples. **B** PCA plot for signal intensity of all control and konzo samples after filtration and SWAN normalization: Principal component analysis done on more than 795,169 probes, reveals similar pattern to before normalization, leading to the assumption that there is not much difference in epigenetic signature between the conditions.

## Data Availability

The datasets generated and analyzed during the current study are available in the GEO repository (GSE180119), https://www.ncbi.nlm.nih.gov/geo/query/acc.cgi?acc=GSE180119.

## Abbreviations

DRC: The Democratic Republic of the Congo
TAN: Tropical ataxic neuropathy
DMP: Differentially methylated probe
SWAN: Subset-quantile within array normalization
GO: Gene ontology
