# Supplementary figures and images for: DNA methylation patterns associated with konzo in Sub-Saharan Africa
Source: Clin Epigenetics. 2022 Dec 19;14:179. doi: 10.1186/s13148-022-01372-x (PMC9764695; doi:10.1186/s13148-022-01372-x)

## Slide 1
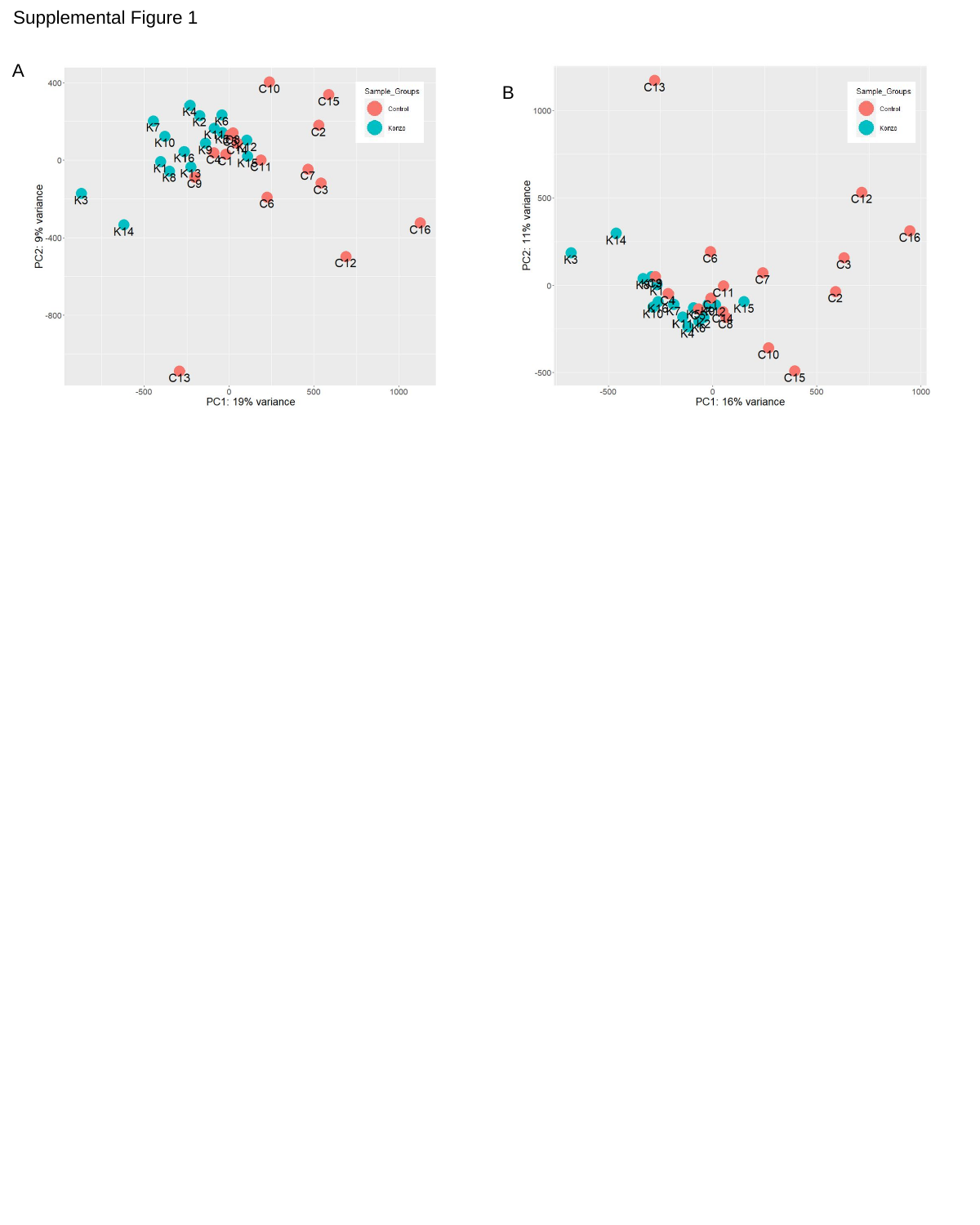

Supplemental Figure 1
A						 		B

Supplement: Supplementary file 2 — Additional file 2: Fig. S1. Principal component analysis (PCA) plots show similarity in signal intensity between konzo and control samples. A PCA plot for signal intensity of all control and konzo samples before normalization: Principal component analysis done on more than 850 K probes (866,087 probes), reveals a tighter overlap of intensities (principal components) between Konzo samples (blue) compared to red (Control) samples. There is some overlap with between the controls and the Konzo sample, which might lead to the assumption that there is not a huge difference in the epigenetic patterns between konzo and control samples. B PCA plot for signal intensity of all control and konzo samples after filtration and SWAN normalization: Principal component analysis done on more than 795,169 probes, reveals similar pattern to before normalization, leading to the assumption that there is not much difference in epigenetic signature between the conditions. [file 13148_2022_1372_MOESM2_ESM.pptx]
